# Supplementary material for: Small-Molecules as Chemiluminescent Probes to Detect Lipase Activity
Source: Int J Mol Sci. 2022 Aug 12;23(16):9039. doi: 10.3390/ijms23169039 (PMC9409280; doi:10.3390/ijms23169039)
Supplement: Supplementary file 1 [file ijms-23-09039-s001.zip › ijms-1859724-Supplementary.pdf]

Supplementary Materials

Small-Molecules as Chemiluminescent Probes to Detect the Lipase Activity

Paolo La Rocca[1], Alessandra Mingione[2], Silvana Casati[2], Roberta Ottria[2], Pietro Allevi[3], Pierangela Ciuffreda[2] and Paola Rota[3,*]

[1] Dipartimento di Scienze Biomediche per la Salute, Università degli Studi di Milano, 20133 Milano, Italy
[2] Dipartimento di Scienze Biomediche e Cliniche, Università di Milano, 20157 Milano, Italy
[3] Dipartimento di Scienze Biomediche, Chirurgiche ed Odontoiatriche, Università degli Studi di Milano, 20133 Milano, Italy; paola.rota@unimi.it (P.R.)

**Table of contents**	Pag.
▪ Chemiluminescence kinetic profile of compounds **2**, **3** and **4**	S1
▪ Figure S2. ^{1}H and ^{13}C NMR of compound **5**	S2
▪ Figure S3. ^{1}H NMR of compound **9**	S3
▪ Figure S4. ^{1}H and ^{13}C NMR of compound **10**	S4
▪ Figure S5. 1H NMR of compound **11**	S5
▪ Figure S6. ^{1}H and ^{13}C NMR of compound **2**	S6
▪ Figure S7. ^{1}H and ^{13}C NMR of compound **3**	S7
▪ Figure S8. 1H and 13C NMR of compound **4**	S8

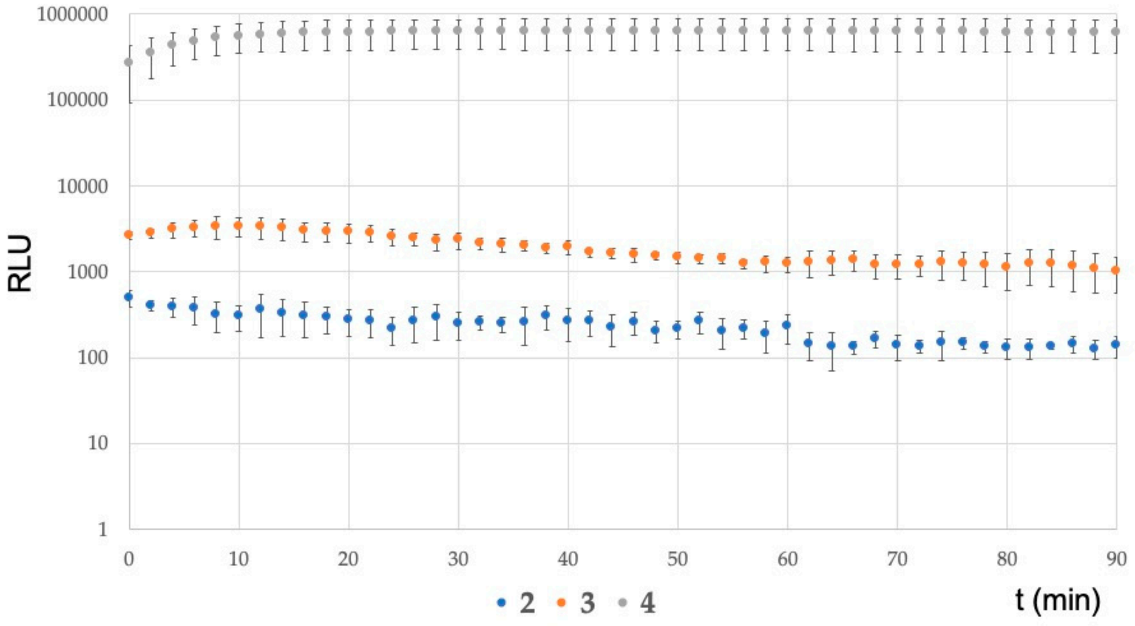

Figure S1. Chemiluminescence kinetic profile of compounds **2**, **3** and **4** in Tris-HCl buffer (pH 7.4, 1mM EDTA, 10% DMSO) in the absence of the enzymes. The compound **4** exhibited a much higher luminescence signal than compounds **2** and **3** in the reaction buffer. The graph shows the mean +/- SD.

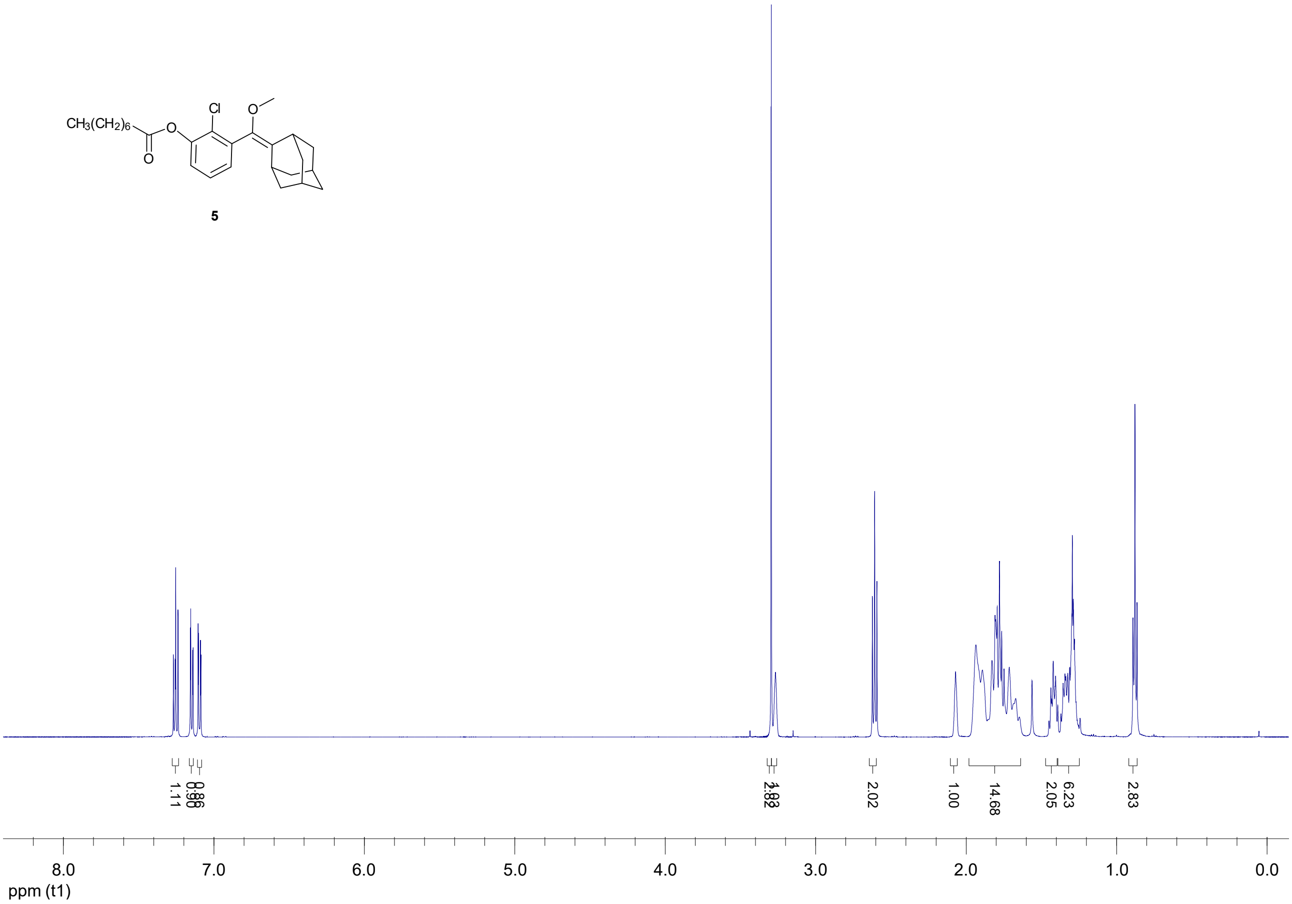

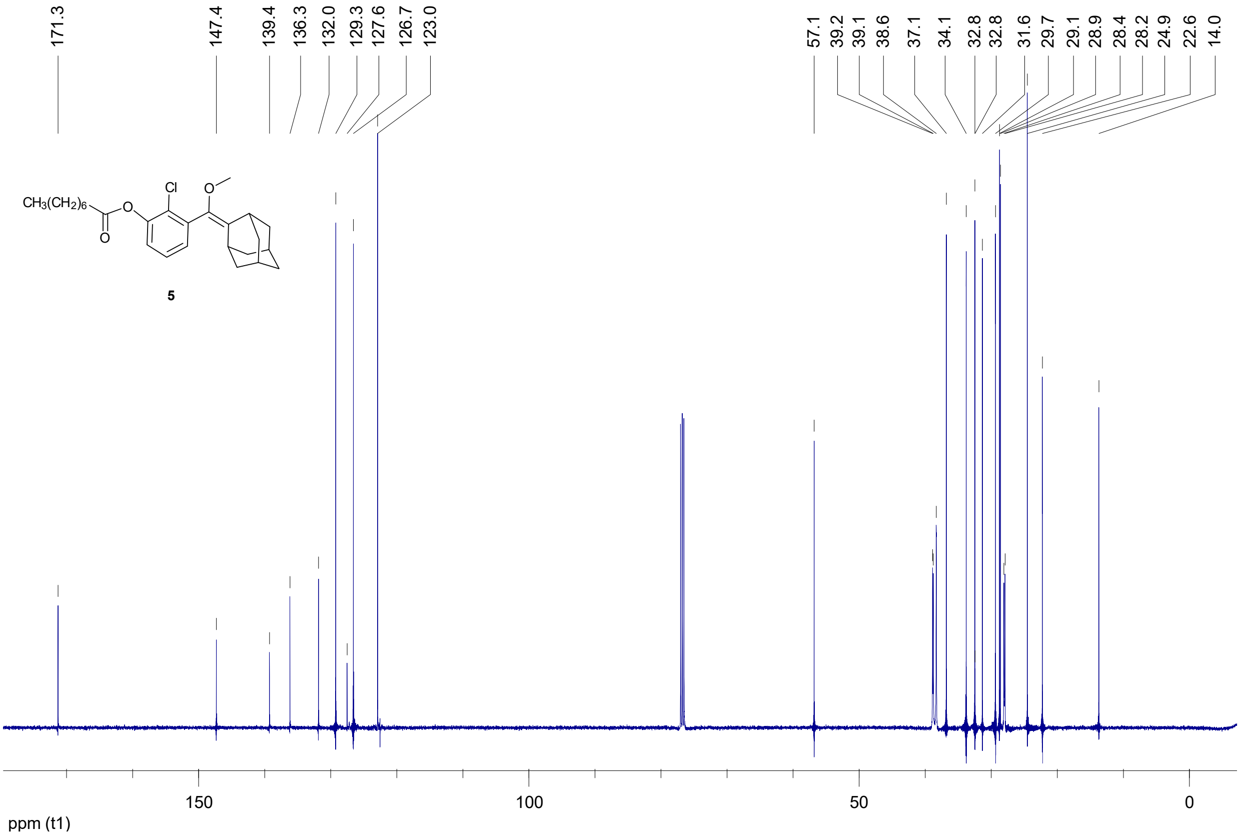

Figure S2. ^{1}H and ^{13}C NMR of compound **5**

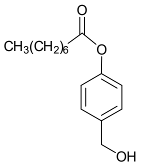

9

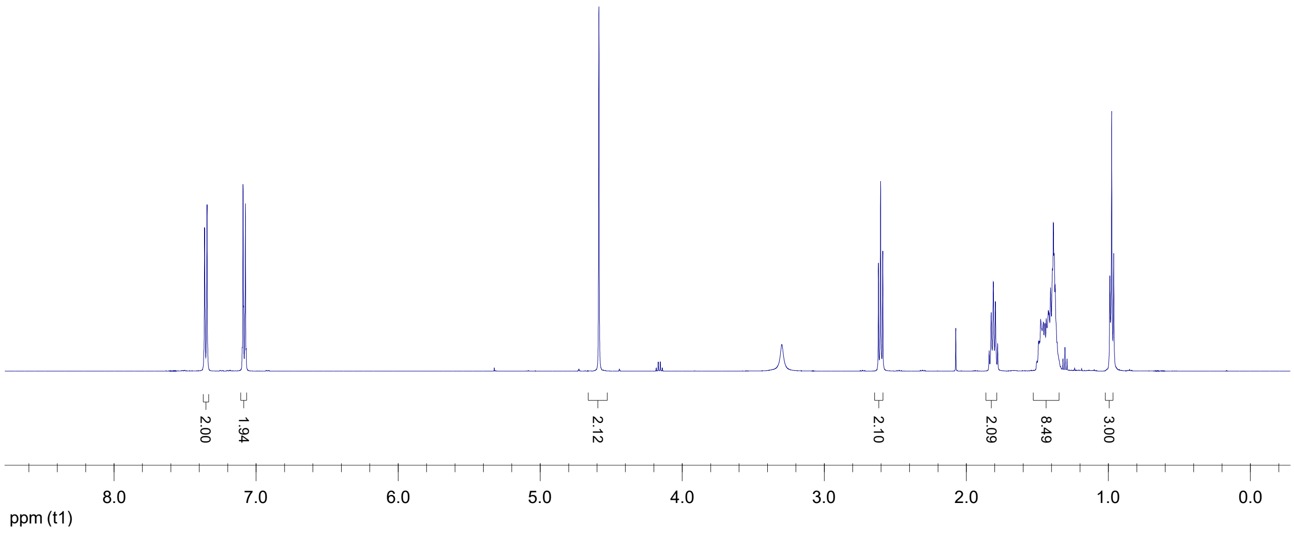

Figure S3. ^{1}H NMR of compound **9**

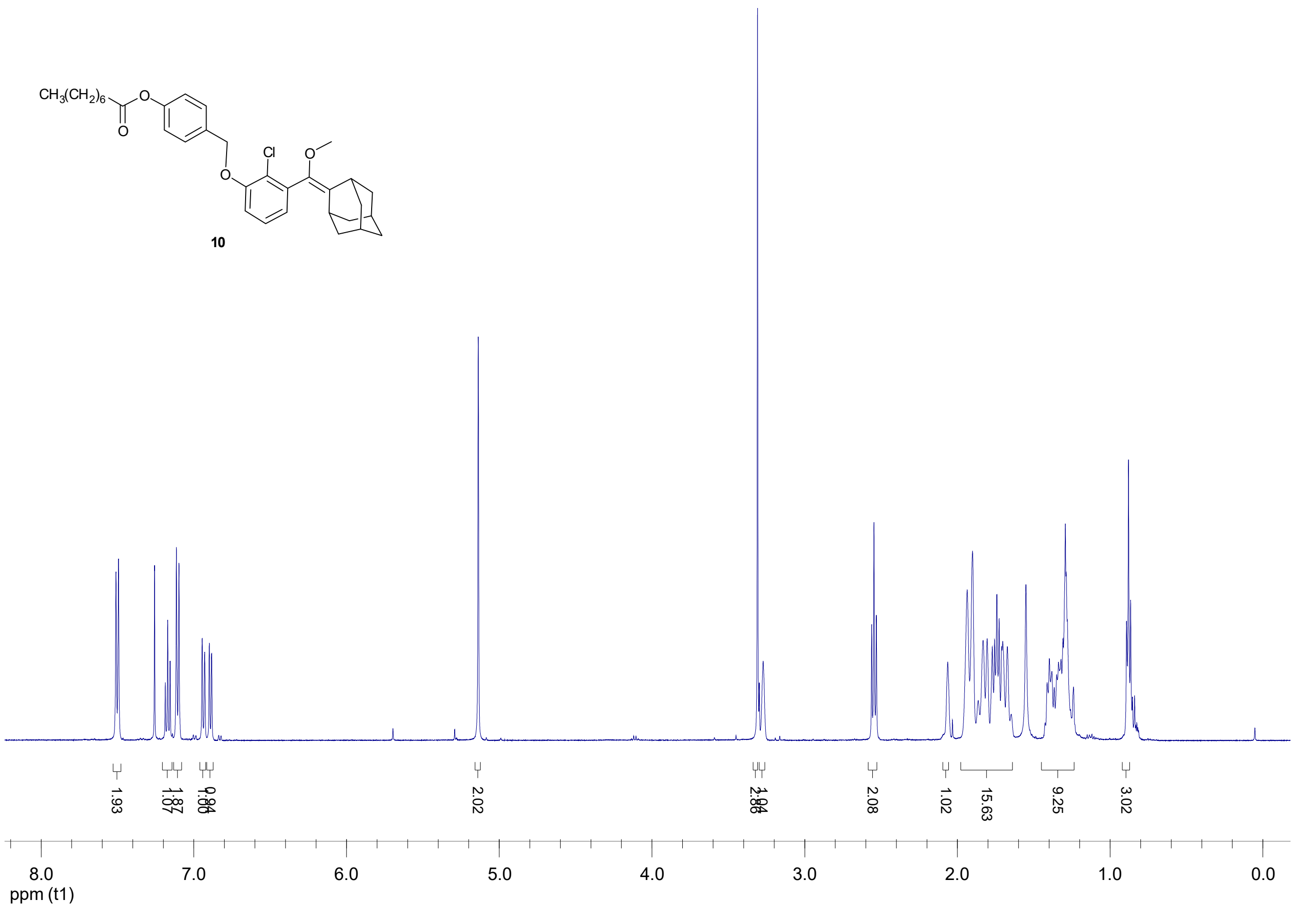

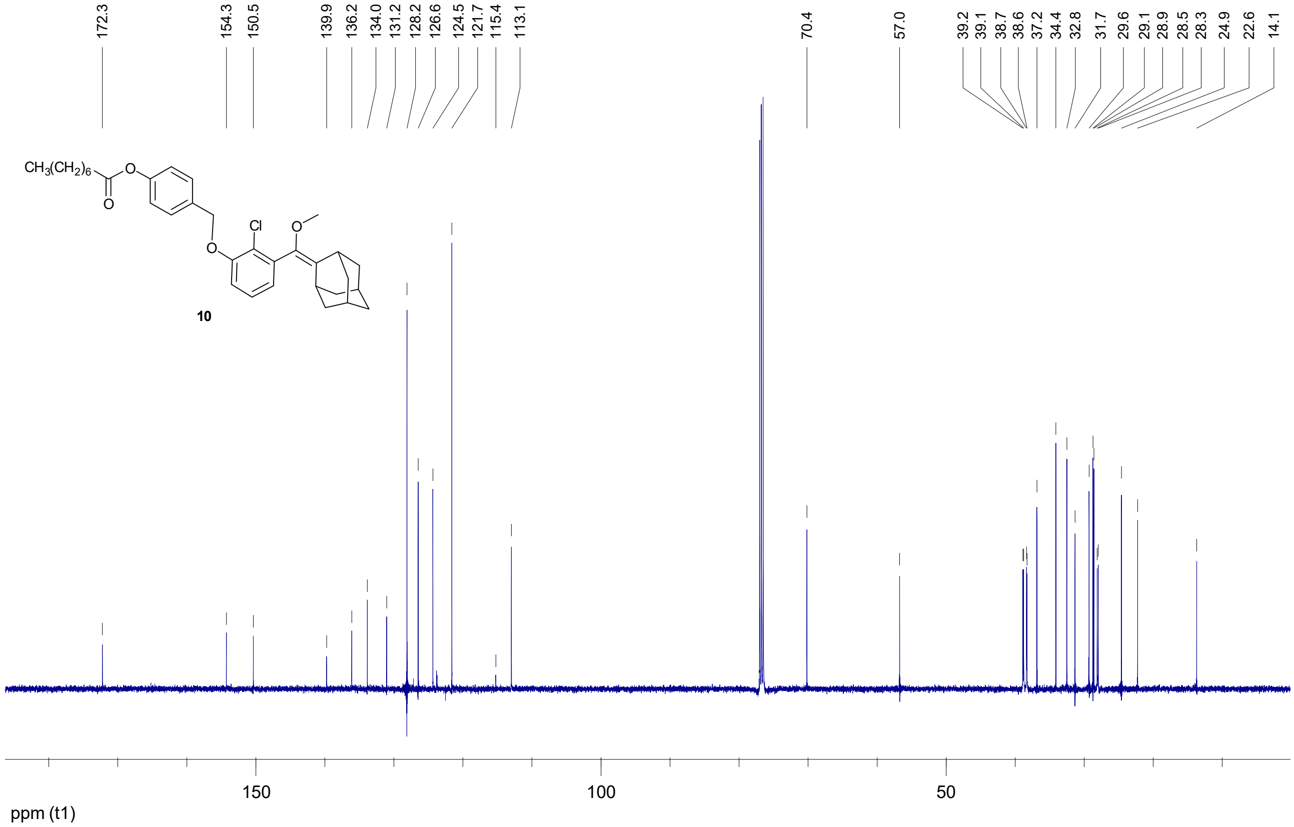

Figure S4. ^{1}H and ^{13}C NMR of compound **10**

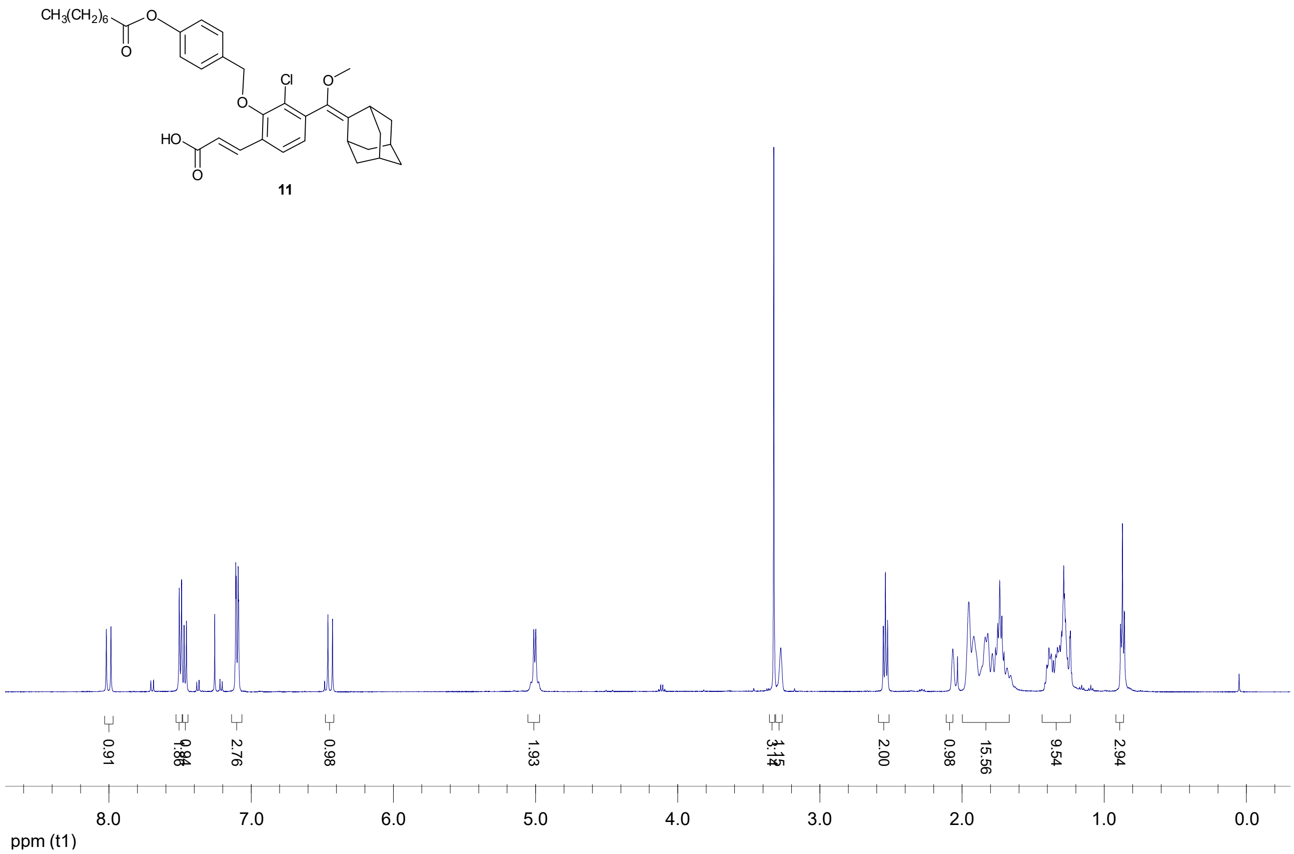

Figure S5. ^{1}H NMR of compound **11**

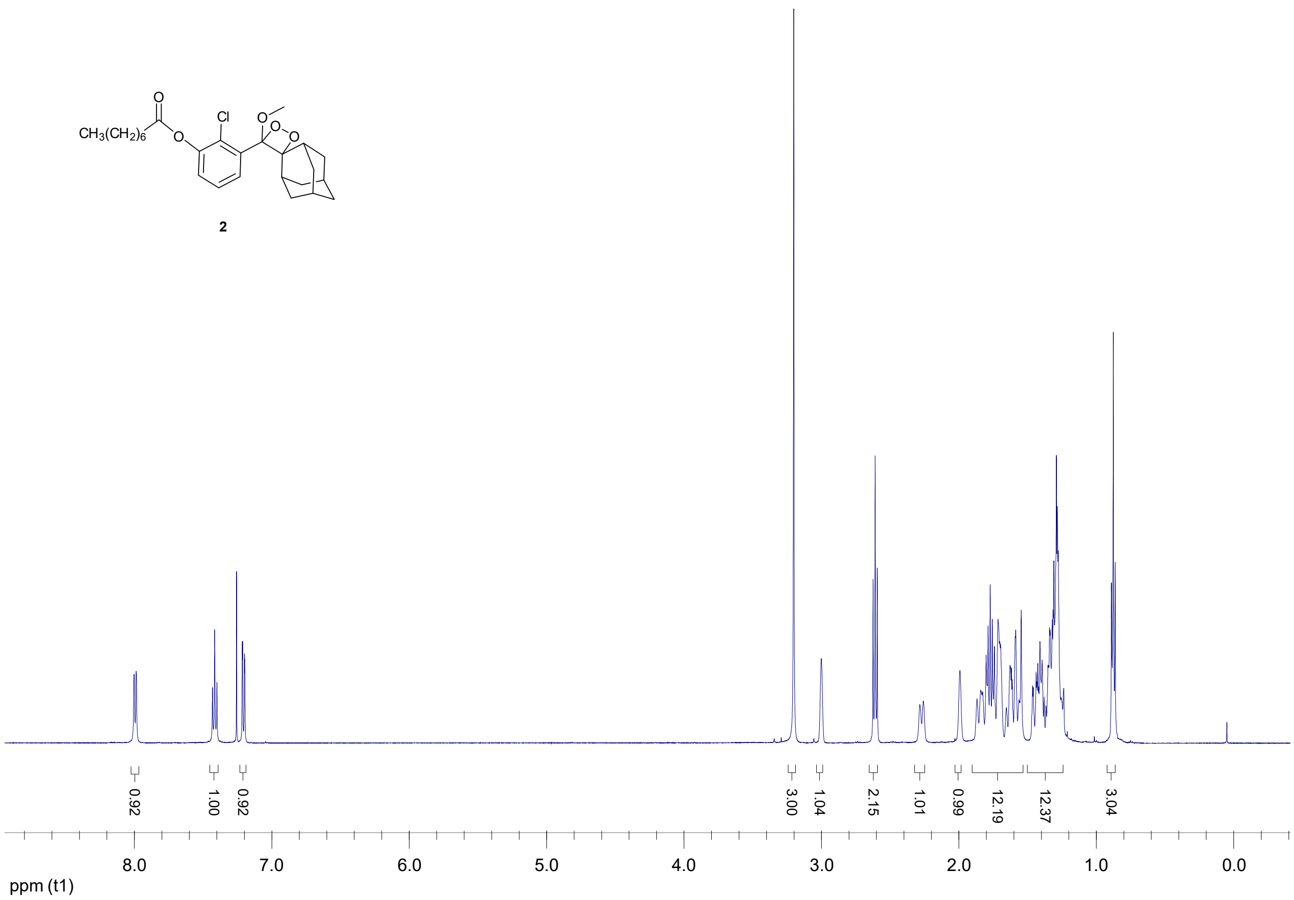

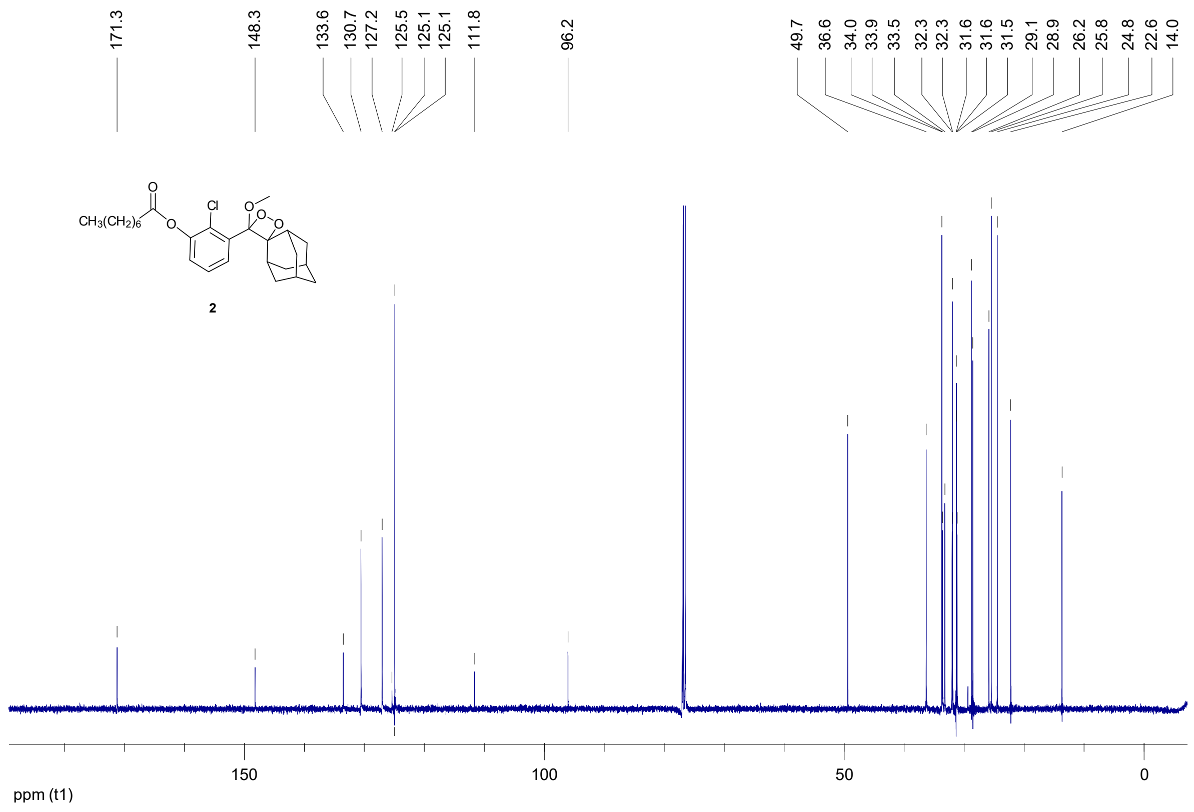

Figure S6. ^{1}H and ^{13}C NMR of compound **2**

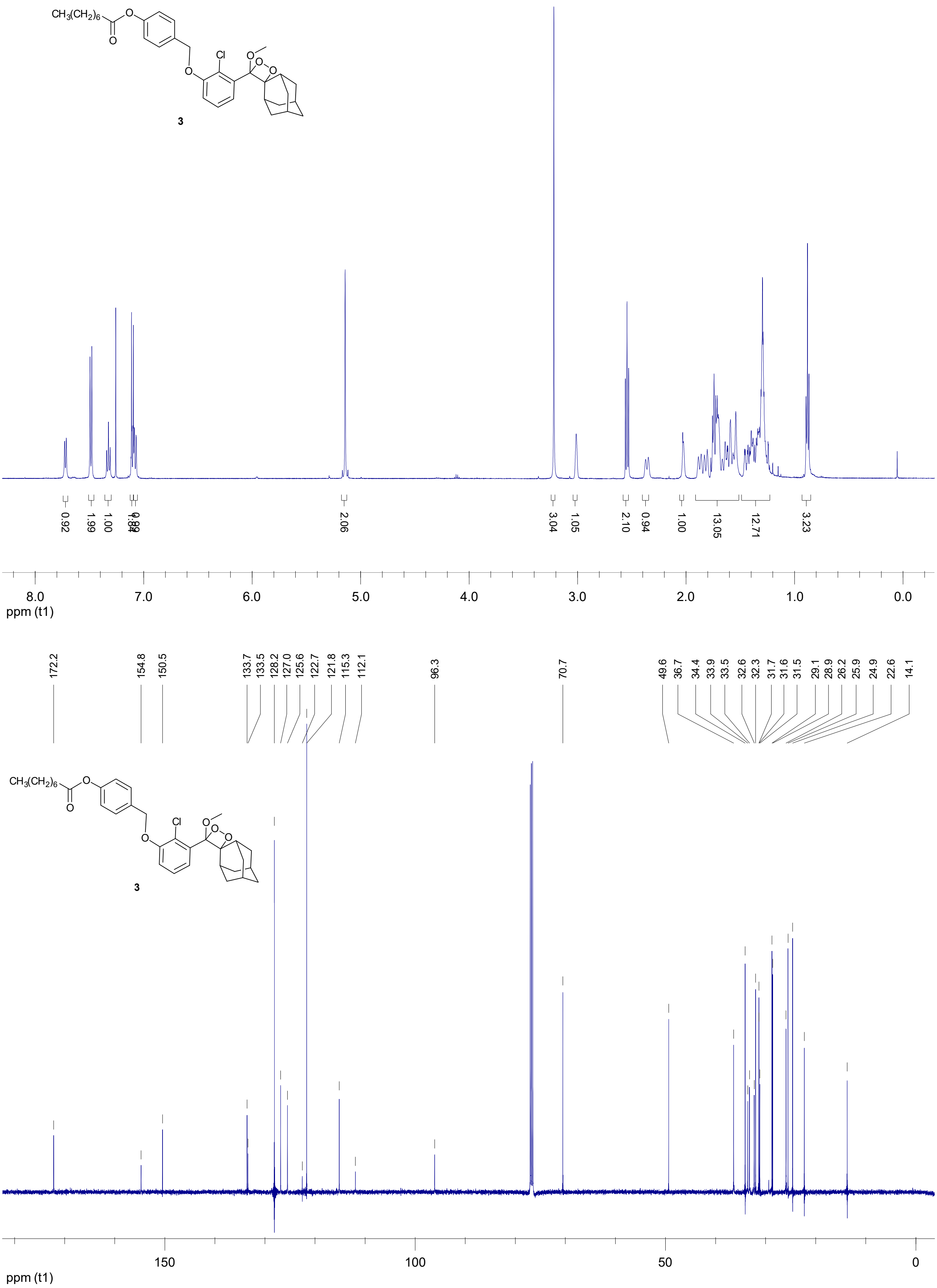

Figure S7. ^{1}H and ^{13}C NMR of compound **3**

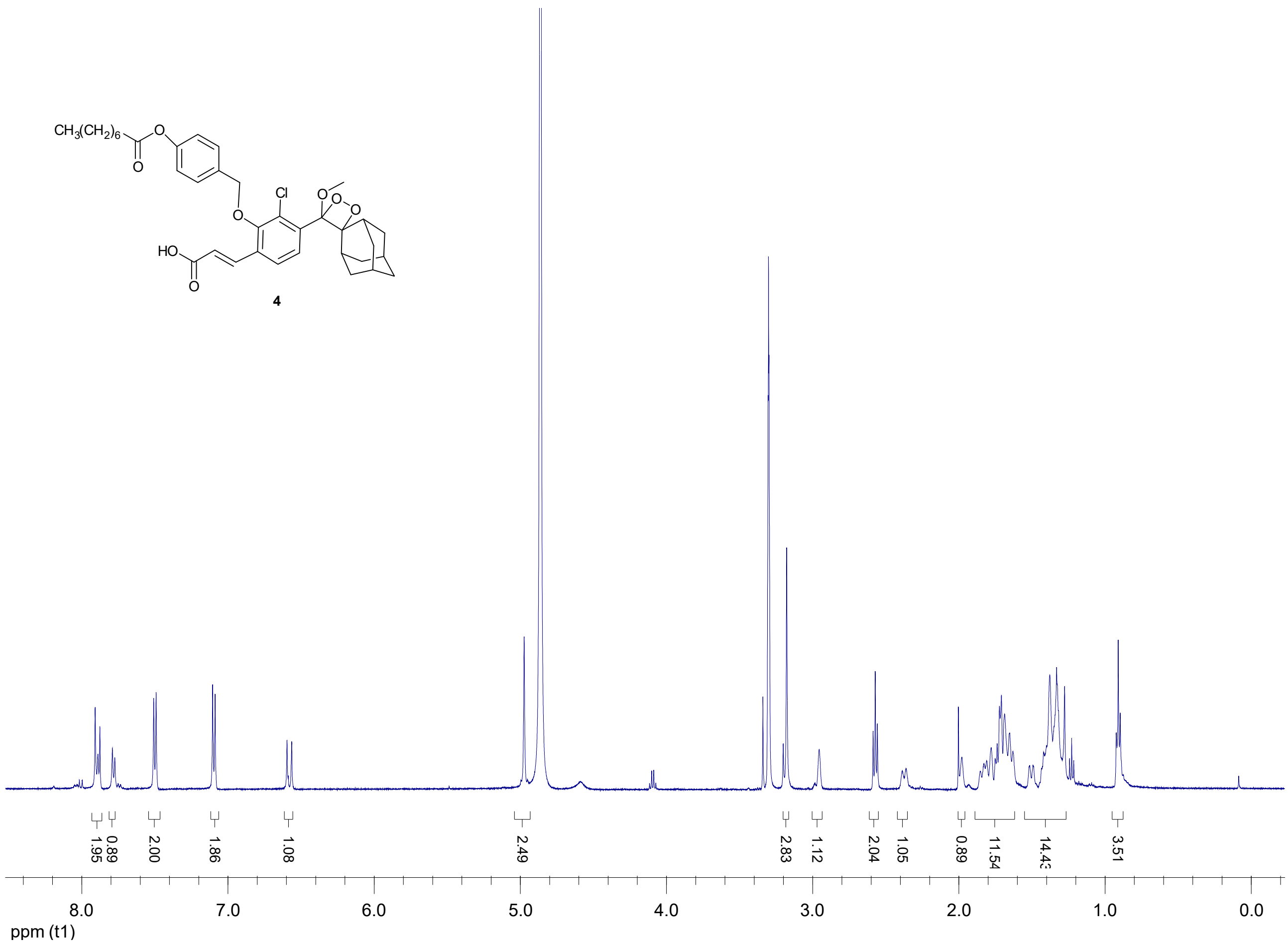

Figure S8. ^{1}H and ^{13}C NMR of compound **4**
